# Synthesis and Biological Activities of a 3′-Azido Analogue of Doxorubicin Against Drug-Resistant Cancer Cells

**DOI:** 10.3390/ijms13033671

**Published:** 2012-03-19

**Authors:** Shuwen Yu, Guisheng Zhang, Wenpeng Zhang, Huanhua Luo, Liyun Qiu, Qingfeng Liu, Duxin Sun, Peng-George Wang, Fengshan Wang

**Affiliations:** 1 School of Pharmaceutical Sciences, Shandong University, Jinan 250012, China; E-Mail: yushuwen@sdu.edu.cn; 2 Department of Chemistry and Biochemistry, The Ohio State University, Ohio 43210, USA; E-Mail: zhangwenpeng@gmail.com; 3 Jinan Central Hospital Affiliated to Shandong University, Jinan 250011, China; E-Mails: zxyylhh@163.com (H.L.); qiuliyun7777@163.com (L.Q.); 4 College of Chemistry and Environmental Sciences, Henan Normal University, Xinxiang 453002, China; E-Mails: zgs@henannu.edu.cn (G.Z.); greendiv@yahoo.com.cn (Q.L.); 5 College of Pharmacy, The University of Michigan, Michigan 48109, USA; E-Mail: duxins@umich.edu; 6 College of Pharmacy, Nan Kai University, Tianjin 300071, China

**Keywords:** anthracycline, Azido, multidrug resistance, ADOX, P-gp

## Abstract

Doxorubicin (DOX), an anthracycline antibiotic, is one of the most active anticancer chemotherapeutic agents. The clinical use of DOX, however, is limited by the dose-dependant P-glycoprotein (P-gp)-mediated resistance. Herein, a 3′-azido analogue of DOX (ADOX) was prepared from daunorubicin (DNR). ADOX exhibited potent antitumor activities in drug-sensitive (MCF-7 and K562) and drug-resistant cell lines (MCF-7/DNR, K562/DOX), respectively. The drug resistance index (DRI) values of ADOX were much lower than that of DOX. The cytotoxicity experiments of ADOX or DOX against K562/DOX, with or without P-gp inhibitor, indicated that ADOX circumvents resistance by abolishing the P-gp recognition. This conclusion was further supported by drug influx/efflux flow cytometry experiments, as well as by molecular docking of ADOX to P-gp. *In vivo* animal tests, ADOX exhibited higher activity and less toxicity than DOX. The current data warranted ADOX for additional pre-clinical evaluations for new drug development.

## 1. Introduction

**Figure 1:**
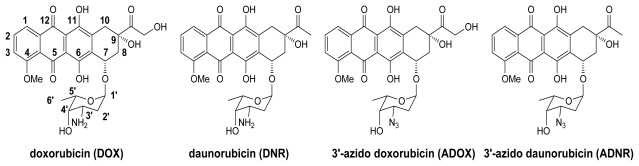
Structure of anthracycline derivatives.

The anthracycline family of antibiotics, such as daunorubicin (DNR) and doxorubicin (DOX) (Figure 1), are among the most effective anticancer drugs used, either as single agents or in combination in chemotherapy. Anthracyclines are widely used in cancer chemotherapy, but the clinical uses are limited by two major problems: multidrug resistance (MDR) [1,2] and cardiotoxicity. MDR is a simultaneous development of resistance to a variety of anticancer drugs in cancer therapy. Many factors are involved in MDR. One of the mechanisms for drug resistance of anthracyclines is mediated by an ABC transporter protein P-glycoprotein (P-gp) because P-gp is overexpressed in many drug-resistant cancer cells [1–8]. P-gp actively exports many anticancer drugs (including anthracyclines) to confer drug resistance. After drugs are diffused into cells, they bind to intracellular domains of P-gp and trigger ATP binding to the nucleoside binding domain (NBD) and the hydrolysis of ATP. The energy from the ATP hydrolysis exports the drugs out of cells. The mechanism of P-gp transport has been well-characterized; however, many attempts to circumvent resistance by P-gp inhibitors have not been successful. The second limitation for the clinical use of anthracyclines is the peculiar, and often irreversible, cardiac toxicity. Cardiac toxicity of anthracyclines is represented by the development of cardiomyopathy and ultimately congestive heart failure. The mechanisms of anthracycline-induced cardiac toxicity are rather complex and multi-factorial. In this paper, our work is based on solving the first problem, drug resistance.

The 3′-NH_2_ group of anthracyclines has been reported to be critical for P-gp binding [9]. Previously, we proved that P-gp mediated MDR of DNR could be successfully overcome via the chemical conversion of DNR into 3′-azido daunorubicin (ADNR) [10,11]. DOX and DNR share the same molecular skeleton except for the side chain at C-14, where DNR carries no functional group and DOX bears a hydroxyl group. Such a small structural difference makes DOX much more potent than DNR, and DOX has broader anticancer activity against leukemia, lymphomas, and various solid tumors including breast, small cell lung, cervical, head, and neck cancers. Thereupon, the strategy to design derivatives of DOX that are not recognized by P-gp is warranted. It is of great interest for us to synthesize the 3′-azido analogue of DOX (ADOX), and to investigate the antitumor activity of it.

## 2. Results and Discussion

### 2.1. Molecular Modeling

**Figure 2:**
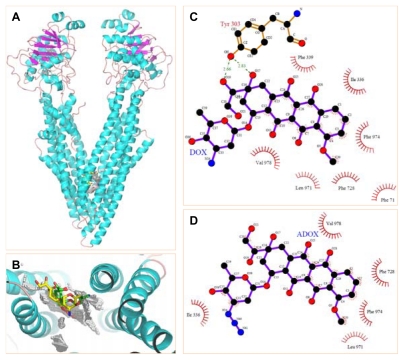
Molecular Docking of DOX and ADOX to P-gp. (**A**) Ribbon diagram of P-gp (PDB entry:3G60) complexed with compounds DOX (yellow sticks) and ADOX (green sticks); (**B**) The proposed docking conformation of compounds DOX (yellow sticks) and ADOX (green sticks) around docking spheres (white spheres) in P-gp; (**C**) Proposed schematic interactions of DOX with P-gp; (**D**) Proposed schematic interactions of ADOX with P-gp.

Before the synthesis of ADOX, we looked at the interactions of DOX and ADOX with human P-gp protein by homology modeling on the basis of X-ray structure of mouse P-gp (PDB entry:3G60) (Figure 2A,B) [12]. In brief, DOX is able to form hydrophobic and/or π-stacking interactions with residues Phe 71, Ile 336, Phe 339, Phe 728, Leu 971, Phe 974 and Val 978 (Figure 2C). Moreover, DOX can contact Tyr 303 by providing two hydrogen bonds. These favorable interactions allowed the docked complex a low binding free energy of −12.70 kcal/mol. In contrast, ADOX bound to P-gp mainly depended on hydrophobic and/or π-stacking interactions with residues Ile 336, Phe 728, Leu 971, Phe 974 and Val 978 (Figure 2D), which granted the proposed complex an unfavorable binding free energy of 1.31 kcal/mol, implying that ADOX was a poor substrate for P-gp. This result may be caused by the bulky azido substitution that would push ADOX away from the active cavity. This modeling result and the broad anticancer spectrum of DOX promoted us to synthesize ADOX for *in vitro* and *in vivo* antitumor tests.

### 2.2. Synthesis of ADOX

**Scheme 1:**
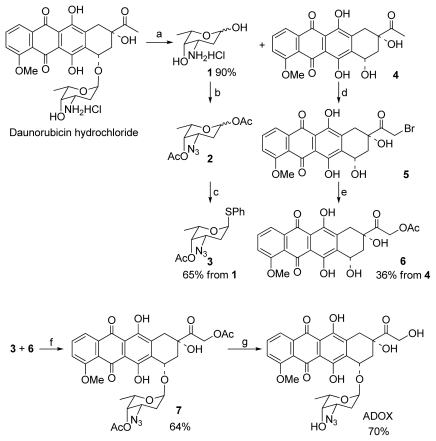
Synthesis of ADOX from DNR. (**a**) 0.2 M HCl, 90 °C, 1 h; (**b**) K_2_CO_3_, CuSO_4_, TfN_3_, solution; then Ac_2_O/pyridine; (**c**) PhSH, BF_3_·Et_2_O, CH_2_Cl_2_, 0 °C, 2 h; (**d**) Trimethylorthoformate, MeOH/1,4-dioxane, rt, 20 min; then Br_2_/CHCl_3_, rt, 6 h; (**e**) NaOAc/acetone, reflux, 6 h; (**f**) NIS, TfOH; (**g**) 0.1 M NaOH/THF, 0 °C.

ADOX was prepared from DNR by employing the glycosylation reaction of 14-actyoxydoxorubicinone (**6**) with an azido sugar (**3**) which was prepared from the hydrolysis of DNR hydrochloride (Scheme 1). The synthesis of aglycon **6** and glycosyl donor **3** started with the hydrolysis of DNR hydrochloride with dilute HCl at 90 °C for 1 h. Aqueous acidic hydrolysis of hydrochloride of DNR with 0.2 M hydrochloric acid at 90 °C gave daunorubicinone (**4**) and hydrochloride of daunosamine (**1**) in 90% yield [13]. Compound **4** was obtained after filtration as a red powder. The filtrate was evaporated to dryness to give the aminosugar **1** containing a little of daunorubicinone **4**. To get a pure **1**, we tried to purify the crude product by recrystallization with ethanol. However, a glycoside, ethyl 3-amino-2,3,6-trideoxy-α-l-*lyxo*-hexoside was obtained in 83% yield instead of hydrochloride of daunosamine. Finally, the pure aminosugar was obtained via a thorough washing of the filtrate with methylene dichloride before the evaporation. The azido sugar **2** could be prepared readily from **1** according to C.-H. Wong’s method [14]. Subsequent treatment of **2** with thiophenol in the presence of boron trifluoride diethyl etherate gave the α-anomer of thioglycoside **3** in overall yield of 65% from aminosugar **1**. The aglycon **6** was directly prepared from its DNR counterpart **4** based on reported procedures [15]. The conversion was accomplished by bromination of **4** with 30% bromine/chloroform in methanol/dioxane followed by the nucleophilic displacement type esterification of 14-bromodaunorubicinone **5** with sodium acetate in acetone. In the literature [15], the yields of these two steps are 92% and 96%, respectively. However, we found the bromination step was in low yield (< 40%) after repeating the procedure several times and the bromide **5** is unstable. We obtained the aglycon **6** in overall yield of 36% from daunorubicinone **4**. In this procedure, the 14-hydroxyl group of doxorubicinone was selectively acetylated which allowed the compound directly use in the subsequent glycosylation. Acetyl group was used for protecting the hydroxyl functions present in glycosyl donor **3** and aglycon **6**, because they were cleavable under 0.1 M NaOH in THF, which allowed the acid and strong base sensitive aglycon moiety in the anthracycline molecule not to be affected in the final deprotection.

Previous research has indicated that the linkage between the sugar moiety and the aglycon is very important. Only the α-linked anthracycline analogues are biologically active. Therefore, the azido sugar has to be introduced stereoselectively in α-linkage. For 2-deoxy sugars, without the stereodirecting ability of 2-substituents, direct and efficient α-selective glycosylations are difficult to realize [16,17]. Hirama and co-workers reported a direct and efficient α-selective glycosylation protocol for the kedarcidin sugar and l-mycarose using AgPF_6_ as a remarkable activator of 2-deoxythioglycosides [18]. Recently, we developed a concise promoter system AgPF_6_/TTBP to activate 2-deoxythioglycosides for synthesis of α-linked glycosides [13,19]. Therefore, in our glycoaylation shown in Scheme 1, glycosyl sulfide was used as glycosyl donor for the α-glycosylation of the azido sugar. Initially, we tried to use the promoter system AgPF_6_/TTBP in the glycosylation. However, we found the promoter system did not work well for the glycosylation of the glycosyl acceptor **6** with donor **3**. Though the condensation exclusively formed the α-product, the yield was very low (< 8% yield). On scrutinizing various promoter systems for activating glycosyl sulfides, finally we were contended with result obtained by NIS/TfOH system. The mixture of aglycon **6** and sugar donor **3**, in the presence of NIS and molecular sieves (4 Å, < 5 microns, freshly activated), was treated for 0.5 h with TfOH at 0 °C to give the glycosylated product **7** in 64% yield (Scheme 1). The ^1^H NMR data indicated that the desired α-linkage was formed predominantly (α:β > 10:1). The ^1^H NMR of **7** showed that the value of *J*_1′,2′_ (2.4 Hz) was in accord with α-anomer. Mild deprotection of **7** with 0.1 M NaOH in THF afforded the final product ADOX in 70% yield. The NMR spectra of the deprotective product were identical to that of the compound ADOX obtained in the procedure shown in Scheme 1.

### 2.3. *In Vitro* Biological Tests of ADOX

**Figure 3:**
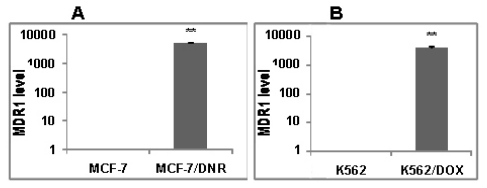
Real-time PCR results of MDR1 mRNA levels. (**A**) Breast cancer cell lines MCF-7 and drug-resistant cell lines MCF-7/DNR; (**B**) Leukemia cell lines K562 and drug-resistant cell lines K562/DOX. Compared with the MDR1 mRNA levels in drug-sensitive cells, that in drug resistant cells were significant higher (** *P* < 0.01).

**Figure 4:**
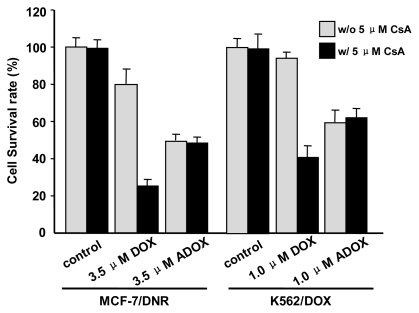
Cytotoxicities of DOX and ADOX in the presence (w/o 5 μM CsA) or absence (w/5 μM CsA) of 5 μM cyclosporine A (CsA) on drug-resistant breast cancer MCF-7/DNR (3.5 μM) and leukemia K562/DOX (1.0 μM) cell lines. This figure is an analysis of the IC_50_ data in table 1. The “control” refers to the absence of DOX/ADOX, CsA alone (5 μM) did not show any cytotoxicity to MCF-7/ADR and K562/Dox.

**Figure 5:**
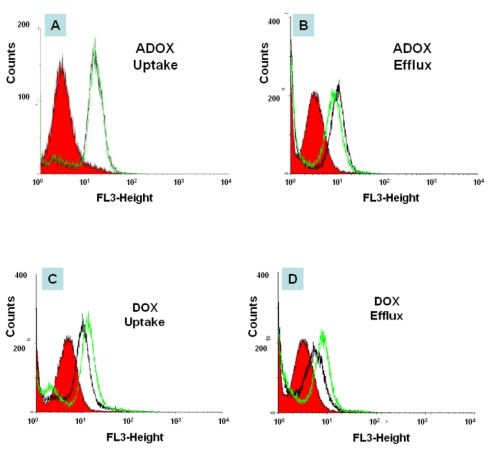
Drug uptake and efflux of DOX and ADOX (2 μM) in drug-resistant leukemia cells (K562/DOX) in the presence (green line) or the absence (black line) of P-gp inhibitor (5 μM CsA) by FACS. Red filled area is control (cell without drug treatment). Black is cell with drug treatment, but without CsA. Green is cell with drug treatment, with CsA.

To test if the new anthracycline analogue ADOX could overcome drug resistance, MTS assays were performed, utilizing MCF-7 human breast cancer cells, K562 human leukemia cells, and their corresponding drug resistance cell lines MCF-7/DNR, K562/DOX. As shown in Table 1, ADOX showed lower IC_50_ values against resistant cell lines MCF-7/DNR (3.5 μM) and K562/Dox (0.87 μM) cells than DOX (20 μM and 27 μM, respectively), whereas ADOX showed higher IC_50_ values against drug-sensitive cell lines MCF-7 (2.2 μM) and K562/Dox (0.64 μM) cells than DOX (0.11 μM and 0.080 μM respectively). Therefore, drug resistance index values (DRI, ratio of IC_50_ in drug-resistant cells over IC_50_ in drug-sensitive cells) of ADOX were 1.6 and 1.4 μM, respectively, which were much smaller than those of DOX. The MTS assay results indicated that, unlike DOX, ADOX could efficiently overcome drug resistance. Real-time PCR data indicated that the resistant cell lines MCF-7/DNR and K562/DOX were up-regulated in the expression of MDR1 (the gene product of MDR1 is P-gp), as compared with MCF-7 and K562 cell lines (Figure 3A,B). The P-gp protein was undetectable in drug-sensitive cell, while P-gp was significantly induced in drug-resistant cell as confirmed by Western blot [20]. This meant that ADOX might overcome drug resistance via avert the P-gp recognition. To prove this hypothesis, P-gp inhibitor cyclosporine A (CsA) was employed in the MTS assays (Figure 4) and FACS experiments (Figure 5). As expected, the addition of CsA to drug-resistant cell lines could enhance the intracellular concentration of ADOX (Figure 5A,B), and in turn lower the IC_50_ values (Figure 4). And the addition of CsA to drug-resistant cell lines had little effect on the retention and cytotoxicities of DOX, which strongly supported that modifications of sugar moiety of anthracycline drug DOX could well overcome P-gp mediated drug resistance.

**Figure 6:**
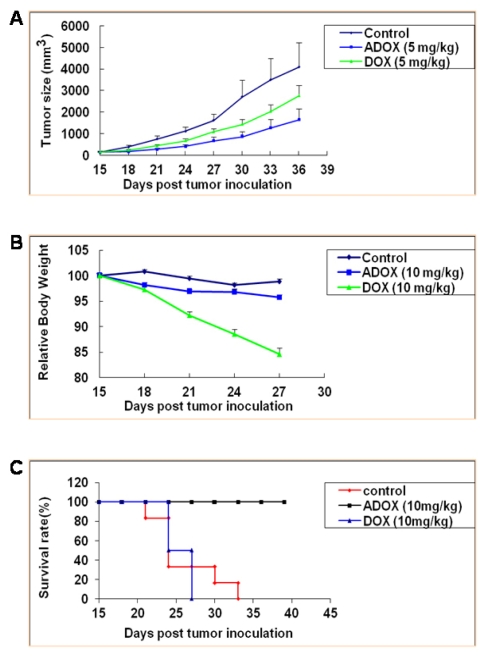
*In vivo* anticancer activity and cytotoxicity of DOX and ADOX in xenograft mice model of drug-resistant leukemia cells (K562/DOX). (**A**) Anticancer activity; (**B**) Relative body weight change; (**C**) Animal survival rate. *N* = 8 for each group. Each point represents the mean ± SE of each group.

To explore the potential of ADOX in clinical applications, *in vivo* efficacy and toxicity experiments were performed. In the xenograft mouse model experiments, 10^7^ drug-resistant leukemia K562/DOX cells were injected subcutaneously into nude mice. After 14 days, the tumor reached greater than 100 mm^3^. From day 15, ADOX (5 or 10 mg/kg) and DOX (5 or 10 mg/kg) were injected in to the mice intraperitoneally twice per week for 3 weeks. The tumor volume was measured every 3 days. As shown in Figure 6, On 15, 18, 21, 24, 27, 30, 33 and 36 days, the average tumor sizes of control group were respectively 158.27, 382.92, 759.99, 1116.13, 1626.12, 2712.98, 3503.27, 4101.17 and 4219.97 mm^3^, while that of DOX group were respectively 158.11, 242.37, 449.16, 678.09, 1097.01, 1433.07, 2031.04 and 2763.80 mm^3^, and that of ADOX group were respectively 141.66, 173.87, 284.57, 417.58, 678.10, 859.14, 1276.01, 1652.39 and 2172.12 mm^3^. Tumor growth was very rapid in the control group (without drug treatment), whereas ADOX and DOX significantly inhibited tumor growth after drug administration. ADOX (5 mg/kg) showed 1.9-fold higher maximum growth inhibition rate against drug-resistant K562/DOX than DOX (Figure 6A), indicating that in vivo efficacy of ADOX was superior to that of DOX.

When ADOX and DOX were given to the xenograft model at the dose of 10 mg/kg twice a week for 3 weeks, the body weight of DOX-treated mice decreased about 18% and all of them died after three-week-administration, while the ADOX treatment group and control group did not show any significant body weight change (Figure 6B). All animals (8/8) in the ADOX group survived (100%) after 50 days, while the mice in the DOX group (6/6) died before 30 days (due to both tumor growth and drug toxicity; 50% of the mice died before 20 days). The mice in the control group (5/5) all died in 33 days (50% of the mice died in 25 days) (Figure 6C). To further test the *in vivo* toxicities of ADOX, mice LD_50_ tests were performed, in which the mice were divided into six groups. The doses of ADOX in the six groups ranged from 120 mg/kg to 20 mg/kg, and the dose ratio of adjacent group was 0.7. After one dose i.p. administration of ADOX, the mice were observed for 14 days. The LD_50_ of ADOX in mice was calculated to be 35 mg/kg (Table 2), which was more than two folds of the reported LD_50_ value of DOX (14 mg/kg) [21]. All these experiments indicated that *in vivo* administration of ADOX could induce less toxicity than DOX.

## 3. Experimental Section

### 3.1. Synthesis

All solvents were dried with a solvent-purification system (Innovative Technology, Inc.). DNR hydrochloride (Greenfield Chemicals, Inc.) and DOX hydrochloride (Hisun Pharma) were directly used without further purification. Analytical TLC was carried out on E. Merck silica gel 60 F_254_ aluminum-backed plates. The 230–400 mesh size silica gel (EMD Chemicals, Inc.) was utilized for all chromatographic purifications. All other reagents were purchased from Aldrich, and were used without further purification. ^1^H and ^13^C NMR spectra and the high-resolution mass spectra were collected at The Ohio State University Campus Chemical instrumentation Center. Elemental analysis was carried out on a Heraeus VarioEL-III C, H, N analyzer. The purity of the title compound ADOX was determined > 96% by HPLC analysis (Agilent 1200 HPLC (XDB-C18 column, 5 μm, 4.6 × 150 mm), Conditions: 0.6 mL/min, CH_3_CN/H_2_O (1:1), λ = 254 nm, *t*_R_ = 8.8 min).

#### 3.1.1. Glycosylation for Preparation of 1′,14-*O*-Diacetyl-3′-azido-DOX(7) (Scheme 1)

Doxorubicinone-14-acetate **6** (1 mmol), molecular sieves 4 Å (800 mg), NIS (1.2 mmol), and **3** (1.2 mmol) was dissolved in CH_2_Cl_2_ and stirred for 1 h at rt. A solution of TfOH (0.1 mmol) in CH_2_Cl_2_ was added and stirred at 0 °C for 30 min. The reaction mixture was diluted with CH_2_Cl_2_ (50 mL), washed with aqueous Na_2_S_2_O_3_ (50 mL), brine (50 mL), dried (Na_2_SO_4_) and concentrated, the crude product was purified by column chromatography to afford compound **7** as a red solid (64%). ^1^H NMR (400 MHz, CDCl_3_) δ13.92 (1H, s, HO-6), 13.34 (1H, s, HO-11), 8.03 (1H, d, *J* = 7.6 Hz, H-1), 7.78 (1H, t, *J* = 8.0 Hz, H-2), 7.37 (1H, d, *J* = 8.8 Hz, H-3), 5.30 (1H, d, *J* = 2.4 Hz, H-1′), 5.20 (2H, m, Ha-14, Ha-10,), 4.96 (1H, m, Hb-14), 4.85 (1H, t, *J* = 6.4 Hz, Ha-8), 4.09 (3H, s, MeO-4), 4.05 (1H, m, Hb-10), 3.92 (2H, m, H-7, H-5′), 2.73 (2H, m, Hb-8, H-4′), 2.24 (2H, m, Ha-2′, H-3′), 2.20 (3H, s, AcO), 2.17 (3H, s, AcO), 2.06 (1H, m, Hb-2′), 1.09 (3H, d, *J* = 6.4 Hz, H-6′). ^13^C NMR (100 MHz, CDCl_3_) δ200.7, 186.9, 186.6, 170.5, 160.9, 156.5, 155.1, 135.6, 135.5, 133.9, 120.9, 119.7, 118.3, 111.4, 111.3, 94.0, 80.6, 70.3, 70.0, 65.9, 64.6, 58.0, 56.6, 54.2, 36.6, 30.4, 29.5, 20.7, 20.4, 16.5. MS ES^+^ *m/z* 692 [M + K]^+^. Anal. calcd for C_31_H_31_N_3_O_13_: C, 56.97; H, 4.78; N, 6.43. Found: C, 56.91; H, 4.86; N, 6.36.

#### 3.1.2. Deprotection of 7 to Title Compound ADOX (Scheme 1)

A solution of DOX analogue **7** (100 mg) in THF (5 mL) was cooled in ice-bath to 0 °C, and then 0.1 M NaOH aqueous solution (70 mL) which was cooled in advance in ice-bath was added. After stirring for 8 h, the reaction mixture was neutralized with 0.1 M citric acid, and extracted with CHCl_3_. The extractions were washed with saturated aqueous NaHCO_3_ solution. Concentration and chromatographic purification on silica gel column (MeOH/CH_2_Cl_2_ 1:100–1:70) provided the product ADOX in yield of 70%. ^1^H NMR (400 MHz, CDCl_3_) δ 13.91 (1H, s, HO-6), 13.14 (1H, s, HO-11), 7.94 (1H, dd, *J* = 7.6 Hz, H-1), 7.69 (1H, t, *J* = 8.0 Hz, H-2), 7.30 (1H, d, *J* = 8.0 Hz, H-3), 5.49 (1H, d, *J* = 4.0 Hz, H-1′), 5.22 (1H, m, H-7), 4.65 (2H, s, H-14), 4.41 (1H, s, HO-9), 3.99 (3H, s, MeO-4), 3.94 (1H, m, H-5′), 3.63 (1H, s, H-4′), 3.49 (1H, m, H-3′), 3.19 (1H, dd, *J* = 1.6 Hz, *J* = 18.8 Hz, Ha-10), 2.93 (1H, d, *J* = 18.8 Hz, Hb-10), 2.25 (1H, m, Ha-8), 2.04 (2H, m, Hb-8, Ha-2′), 1.82 (1H, m, Hb-2′), 1.24 (3H, d, *J* = 6.4 Hz, H-6′). ^13^C NMR (100 MHz, CDCl_3_) δ 213.1, 186.8, 186.5, 160.9, 155.8, 155.3, 135.5, 135.2, 133.1, 133.0, 120.7, 119.6, 118.3, 111.4, 111.3, 100.3, 69.5, 69.1, 66.9, 65.1, 56.4, 35.2, 33.7, 28.1, 16.5. MS ES^+^ *m/z* 592 [M + Na]^+^. Anal. calcd for C_27_H_27_N_3_O_11_: C, 56.94; H, 4.78; N, 7.38. Found: C, 56.90; H, 4.88; N, 7.30.

### 3.2. Biology

#### 3.2.1. Cell Culture

Drug-sensitive leukemia cells K562, breast cancer cells MCF-7 and drug-resistant leukemia cells K562/DOX, breast cancer cells MCF-7/DNR were gifts from J. P. Marie (Institute National de la Sante et de la Recherche Medicale, E9912, University of Paris 6, Paris, France). The cells (2,000–10,000) were cultured in RPMI 1640 supplemented with 10% fetal bovine serum (FBS), 1% non-essential amino acid and Penicillin (100 units/mL)/Streptomycin (100 μg/mL) in a humidified atmosphere of 5% CO_2_ and 95% air at 37 °C. Culture medium was changed every 2 to 3 days. Before each experiment, K562/DOX cells were stimulated with 0.1 μM DOX at least for one week and then cultured for 10 days without DOX stimulation. It was assured that P-gp expression level was similar in every experiment.

#### 3.2.2. MTS Assay of ADOX

Drug-sensitive and drug-resistant cells (2,000–10,000) were seeded in 96 well plates in RPMI-1640 and incubated for 24 h. The exponentially growing cancer cells were incubated with various concentrations of compounds for 72 h at 37 °C (5% CO_2_, 95% humidity). After 72 h incubation, tetrazolium[3-(4,5-dimethythiazol-2-yl)]-5-(3-carboxymethoxyphenyl)-2-(4-sulfophenyl)-2*H*-tetrazolium, inner salt (MTS, final concentration, 2 mg/mL) and phenazine methosulfate (PMS, final concentration 25 μM) were mixed and added directly to the cells. After incubated for 3 h at 37 °C, the absorbance of formazan (the metabolite of MTS by viable cells) was measured at 490 nm. The IC_50_ values of the compounds for cytotoxicity were calculated by WinNonlin software [22] from the dose-response curves.

#### 3.2.3. RNA Extraction and Real-Time PCR Analysis

RNA was extracted from cells using Trizol reagent (Invitrogen, Carlsbad, CA, USA) according to the manufacturer’s instructions. Real-time PCR was performed using SYBR Green as the dye. The MDR1 mRNA expression level in each cell line is calculated against β-actin as a control. The MDR1 level in K562/DOX was calculated using K562 as control.

#### 3.2.4. Flow Cytometry (FACS)

The assay was performed on a Becton-Dickinson FACS caliber (San Jose, CA, USA) equipped with an ultraviolet argon laser (excitation at 488 nm, emission at 530/30 and 570/30 nm band-pass filters). Analysis was stopped on acquisition of 50,000 cells. Log fluorescence was collected and displayed as single parameter histograms. Cell media was replaced with fresh 10% FBS RMPI 1640 containing no P-gp inhibitor Cyclosporine (CsA), 60 min before experiment. The cells were centrifuged (1000 rpm for 3 min) at room temperature and resuspended in 10% FBS RMPI1640. 10 million cells (in 50 μL) were transferred to plastic tubes containing 1.95 mL of incubation media with DOX or ADOX. In the uptake phase, cells were incubated with 2 μM DOX or ADOX in 10% FBS RMPI1640 in the presence or absence of 5 μM CsA for 30 min at 37 °C. After centrifugation (1000 rpm for 3 min at 4 °C), the cells were separated into equally two tubes. One tube was washed once with ice cold RMPI (no FBS) and transferred to FACS tube in the staining buffer on ice. These cells represent drug uptake phase. The other tubes were reincubated in 10% FBS RMPI1640 in the presence or absence of 5 μM CsA for an additional 30 min at 37 °C. This represents the drug efflux phase. After final centrifugation (1000 rpm for 3 min at 4 °C), the supernatant was removed. The cells were washed once with cold RMPI 1640, and the cell suspension in staining was transferred to FACS tubes. The drug accumulation in the cells was analyzed by FACS.

#### 3.2.5. Anticancer Activity of ADOX Against Drug-Resistant Cancers in Xenograft Model

Female, 5–7-week-old athymic nu/nu mice were purchased from the Charles River laboratories. Cells (1 × 10^7^) were injected subcutaneously into the right flanks of the mice. Mice bearing tumors of 100–300 mm^3^ in volume (usually 10–15 days after tumor inoculation) were randomized into eight mice per group. Mice were treated with: (1) vehicle alone; (2) DOX at 5 or 10 mg/kg, i.p. twice per week for six injections; or (3) ADOX at 5 or 10 mg/kg, i.p. twice per week for six injections. Tumor volume (V) was recorded every 3 days as V = 0.5 *l w*^2^, where *l* and *w* are the longest and shortest diameters of the tumor mass (in mm), respectively. Body weight was also recorded every 3 days to monitor the toxicity of the treatment. The statistical significance was analyzed using Student’s *t* test, and the differences were considered significant at *P* < 0.05.

#### 3.2.6. Acute Toxicity of ADOX Determined by Mouse LD_50_ Test

Balb/c mice (half male and half female, weighing between 18–22 gram), were obtained from Center for New Drug Evaluation in Shandong University, China. Mice were randomly divided into 6 groups, and the corresponding dose levels were 120, 84, 59, 41, 29 and 20 mg/kg respectively. There were five male and five female mice in each group. The mice were observed for 14 days after one-dose-administration of ADOX, i.p. Numbers of dead mice in each group were recorded (Table 2), and LD_50_ value was determined by using software Origin 7.5 [23].

### 3.3. Computation

#### 3.3.1. Molecular Docking of DOX and ADOX to P-gp

The crystal structure of P-gp (PDB entry:3G60) was chosen for molecular docking. Polar hydrogen atoms were added, and Kollman-all charges were assigned. Hydrogen atoms were added to the 3D ligand structures and all atoms were assigned with AM1-BCC partial charges. The position and conformation of each compound were optimized first by the anchor fragment orientation and then by the torsion minimization method implemented in the DOCK 6 program [24]. Fifty conformations and a maximum of 100 anchor orientations for each compound were generated, and all of the docked conformations were energy minimized by 100 iterations following procedures as described in literature. The docked molecules were ranked based on the sum of the van der Waals and electrostatic energies implemented in the DOCK 6 program.

#### 3.3.2. Statistics

All values are expressed as means ± SD. When appropriate, statistical significance (defined as *P* < 0.05) was determined by Student’s *t* test.

## 4. Conclusions

In this study, a new DOX derivative, ADOX, was synthesized which bore an azido group at the C-3 position of the sugar moiety. ADOX could be conveniently prepared from DOX via one pot diazo transfer reaction in moderate yield as described previously [10]. In this study, ADOX was prepared from DNR which was much cheaper than DOX. ADOX exhibited potent anticancer activity both in drug-sensitive tumor cells (MCF-7 and K562), and in drug-resistant cells (MCF-7/DNR and K562/DOX). Although ADOX proved less effective in drug-sensitive K562 cells than DOX, it was much more effective than DOX in drug-resistant cells, which was helpful to overcome the disadvantage of MDR. Its DRI values were less than 2, which were over 100-fold lower than those of DOX. Considering the dramatic high level of MDR1 mRNA levels in these two drug resistant cell lines, we hypothesized the small DRI values of ADOX were attributed to its abolishment of P-gp recognition. This was supported by the MTS experiments and FACS experiments with the addition of P-gp inhibitor CsA. The molecular modeling using 3G60, a mouse homolog to human P-gp [12], were performed to prove that ADOX was a much weaker binding substrate to P-gp than DOX. The computational binding free energy of ADOX to P-gp was 1.31 kcal/mol, higher than that of DOX (−12.70 kcal/mol).

*In vivo* efficacy and toxicity tests were carried out. Compared with DOX, ADOX was more effective in shrinking tumor volume size in drug-resistant K562/DOX xenograft mice model experiments via consecutive i.p. administration of 5 mg/kg dose every three days. At the same time, ADOX was less toxic than DOX. The mice LD_50_ value of ADOX was determined to be 35 mg/kg, approximately double the reported data of DOX (14 mg/kg) [21]. The current *in vivo* assay results proved ADOX as a promising drug candidate, and warranted it for further pre-clinical evaluations.

## Figures and Tables

**Table 1:** The cytotoxicities (IC_50_ μM) of DOX and ADOX (MTS Assay).

Cell lines and DRI a	DOX	ADOX
MCF-7 cell line	0.11	2.2
MCF-7/DNR cell line b	20	3.5
DRI^a^	182	1.6
K562 cell line	0.080	0.64
K562/DOX cell line b	27	0.87
DRI a	338	1.4
CV-1 cell line c	25	2.8
1.5 μM-CPT-CV-1 cell line d	> 10	6.6
4 μM-CPT-CV-1 cell line d	> 10	2.6
3 μM-mAMSA-CV-1 cell line d	> 10	2.4

a DRI is the abbreviation for drug resistant index, which is equal to the ratio of IC_50_ in drug-resistant cells (MCF-7/DNR and K562/DOX) over IC_50_ in drug-sensitive cells (breast cancer MCF-7 cells and leukemia K562 cells);

b Drug resistant cell lines;

c CV-1 is African green monkey kidney cell line;

d 1.5 μM CPT-CV-1, 4 μM CPT-CV-1 and 3 μM mAMSA-CV-1 were drug resistant cell lines that were selected stepwise for camptothecin or mAMSA resistance. They were finally maintained in 1.5 μM camptothecin, 4 μM camptothecin and 3 μM mAMSA, respectively.

**Table 2:** Mouse LD_50_ test of ADOX.

Dose (mg/kg) a	120	84	59	41	29	20
Number of mice in test b	10 c	10	10	10	10	10
Number of dead mice	9	7	6	6	4	0

a LD_50_ value of ADOX was 35 mg/kg, which was determined by using software Origin 7.5;

b The mice were observed for fourteen days after one-dose-administration of ADOX, ip;

c There were five male and five female Balb/c mice in each group, and they weighed between 18–22 gram.
